# Innate Immune Mechanisms and Immunotherapy of Myeloid Malignancies

**DOI:** 10.3390/biomedicines9111631

**Published:** 2021-11-06

**Authors:** Sara Small, Yazan Numan, Leonidas C. Platanias

**Affiliations:** 1 Robert H. Lurie Comprehensive Cancer Center, Northwestern University, Chicago, IL 60611, USA; yazan.numan@nm.org (Y.N.); l-platanias@northwestern.edu (L.C.P.); 2 Division of Hematology-Oncology, Department of Medicine, Feinberg School of Medicine, Northwestern University, Chicago, IL 60611, USA; 3 Department of Medicine, Jesse Brown Veterans Affairs Medical Center, Chicago, IL 60612, USA

**Keywords:** immunotherapy, AML, interferon, antibodies, myeloid malignancies, CAR-T

## Abstract

Similar to other cancers, myeloid malignancies are thought to subvert the immune system during their development. This subversion occurs via both malignant cell-autonomous and non-autonomous mechanisms and involves manipulation of the innate and adaptive immune systems. Multiple strategies are being studied to rejuvenate, redirect, or re-enforce the immune system in order to fight off myeloid malignancies. So far, the most successful strategies include interferon treatment and antibody-based therapies, though chimeric antigen receptor (CAR) cells and immune checkpoint inhibitors are also promising therapies. In this review, we discuss the inherent immune mechanisms of defense against myeloid malignancies, currently-approved agents, and agents under investigation. Overall, we evaluate the efficacy and potential of immuno-oncology in the treatment of myeloid malignancies.

## 1. Introduction

The human immune system is capable of recognizing both foreign organisms and altered versions of self. This latter ability is a key mechanism that affords protection against neoplasms. Efficacious innate and adaptive immune systems are essential for this protection to succeed, whereas a dysfunctional immune system allows cancer cells to grow unhindered [1]. The recognition of the immune surveillance’s role in preventing neoplastic growth and the discovery of immune system manipulation by malignant cells has led to the utilization of immune-targeted strategies in multiple malignancies. The launching of these novel strategies marked a new era of immuno-oncology in cancer therapeutics [2,3]. One strategy in immuno-oncology involves harnessing the power of the natural immune system to increase recognition and eradication of tumors; for example, by removing road blocks to this process with checkpoint inhibitors, ramping up the immune response with interferon treatment [4] or cancer vaccines, or directing immune cells to attack neoplastic cells with antibody therapy. Other strategies include introducing exogenous components of immunity such allogeneic hematopoietic stem cell transplants (allo-HSCT), donor lymphocyte infusions, and chimeric antigen receptor T-cell (CAR-T) cell therapy [5].

Allo-HSCT represents the oldest and most intensive form of immunotherapy against myeloid malignancies, to date, in the form of the graft-versus-leukemia effect [6,7]. For many patients, such as those with secondary AML, allo-HSCT represents the only modality of potential cure [8]. Other immune-related treatments, currently approved for myeloid malignancies, include the antibody-drug conjugate gemtuzumab-ozogamicin (GO) [9]. Additional agents that have shown promise include Interferon alfa (IFNα), unconjugated antibodies, multivalent antibodies, and antibody-drug conjugates [10]. Checkpoint inhibitors, which have been successful in some solid tumors, and CAR-T cells, which have activity in lymphoid malignancies, are currently under investigation for use in myeloid malignancies. There are several useful previous reviews describing immunologic agents under investigation for treatment of AML [11,12,13,14,15,16,17]; in this review, we update relevant information in the field and also delve into the innate mechanisms of defense against myeloid malignancies, as well as interferon’s potential role in treating some of these malignancies.

## 2. Innate Immune Cells in the Defense against Myeloid Malignancies

The cells of the innate immune system are poised to detect various cellular alterations and affect responses directly, as well as indirectly, by activating the adaptive immune system. These alterations include those related to microbial infections as well as markers of stressed cells, such as DNA damage, which can direct innate cells to tumor cells [1]. Three essential components of the innate immune system involved in preventing neoplastic growth are natural killer (NK) cells, dendritic cells, and macrophages.

### 2.1. NK Cells

NK cells are able to recognize foreign elements or altered self and respond directly through various mechanisms. One mechanism, called antibody-dependent cellular cytotoxicity (ADCC), requires the binding of an antibody to the target cell. The CD16a receptor on the NK cell then binds to the fraction crystallizable (Fc) portion of the antibody and triggers degranulation of granzymes and perforin, causing lysis of the target cell [18]. NK cells also trigger caspase-dependent apoptosis of target cells by stimulating death receptors on the target cell’s surface [19]. Importantly, DNA damage, a hallmark of tumorigenesis, can upregulate NK ligands on tumor cells [20]. There are multiple preclinical studies showing the ability of NK cells to kill acute and chronic myeloid leukemia cells [21,22,23]. The expression of NK-activating ligands on AML blasts is linked to positive outcomes in AML patients undergoing chemotherapy [24], and genetic knockout of key NK cell receptors increases tumor formation in mice [25]. There is also evidence that progression of AML, MDS, and CML is inversely correlated with the number and function of NK cells [26]. CAR-NK cells are a novel avenue of treatment and are discussed below.

### 2.2. Dendritic Cells

Dendritic cells are another essential cell type in the innate immune system’s defense against malignancies. They are antigen-presenting cells that connect the innate and adaptive immune systems, e.g., by activating anti-tumor T cells. In the context of malignancy, dendritic cells are activated by tumor-derived DNA and other damage-associated molecular patterns (DAMPs) and pathogen-associated molecular patterns (PAMPs) via their expression of pattern recognition receptors [27,28]. Once activated, dendritic cells upregulate expression of cytokines, MHC receptors, and co-stimulatory molecules, and they migrate into lymph nodes where they are thought to display their antigen and prime T cells [29]. Since dendritic cells are able to stimulate cytotoxic T cells, there are multiple dendritic cell-based vaccines in development, utilizing leukemia-associated antigens, though these vaccines are not yet being used in clinical practice [30,31,32].

### 2.3. Macrophages

Macrophages play a key role in normal tissue homeostasis and inflammatory responses to infections [33]. There are two main subtypes of macrophages, termed M1-like and M2-like. M1-like macrophages are “classically activated” (via LPS, IFNγ, or GM-CSF) and have a pro-inflammatory and anti-tumor phenotype [33]. In contrast, M2-like macrophages are “alternatively-activated” (via IL-4), and they have an immunosuppressive and pro-tumor phenotype [33]. Both types of macrophages can infiltrate tumors, where they become tumor associated macrophages (TAMs). Interestingly, in some studies, tumor infiltration with M1-like macrophages corresponds to a favorable prognosis, whereas infiltration with M2-like macrophages correlates with a poor prognosis; in general, the number of M1-like cells tends to decrease during tumor progression [34,35]. M1-like TAMs can eliminate cancer cells by driving T cell proliferation (both cytotoxic T lymphocytes and T helper 1 cells), secreting the toxic molecule nitric oxide (NO) and pro-inflammatory cytokines (e.g., IFNγ, IL-1β, IL-12, and TNFα) [36]. IFNγ signaling inhibits angiogenesis and causes cancer cells to upregulate MHC class I receptors, making them more vulnerable to MHC-restricted killing. Unfortunately, cancer cells that have been exposed to IFNγ also upregulate molecules such as PD-L1 and indoleamine 2,3 dioxygenase 1 (IDO1) that cause T cell exhaustion and allow tumor progression [36].

Multiple therapies, utilizing the innate ability of macrophages to suppress cancer growth, are under investigation. For example, inhibition of the macrophage checkpoint CD47/SIRPα is a novel and promising strategy for treatment of AML and MDS, and it is discussed in detail below [37]. Orchestrating a switch from M2-like to M1-like phenotype in TAMs is also a possible therapeutic strategy [36]. For example, one preclinical study found that paclitaxel causes tumor regression by shifting the balance of macrophages from the M2-like phenotype to the M1-like phenotype [38]. Additionally, in another preclinical study, an HDAC inhibitor that induces macrophages, to assume an M1-like phenotype, causes tumor regression in a macrophage-dependent manner [39].

Despite the cancer-preventing abilities of NK cells, dendritic cells, and macrophages, along with other innate immune cells and the adaptive immune system, not all tumor cells are cleared by the immune system, resulting in “immune-edited” tumor cells that are able to proliferate freely. Nevertheless, many successful therapies against myeloid malignancies utilize the immune system in some manner.

## 3. Interferon in Myeloid Malignancies

One of the most important families of genes regulated by the innate immune system is the interferon family. Interferons are cytokines that have multiple functions in infection and cancer [27,40,41,42]. There are two classes and multiple subtypes of interferons, but for the purposes of this review, we will focus on the type I interferon interferon-alpha (IFN-α), which is the most clinically-relevant interferon. IFN-α binds to the IFN-α receptor (IFNAR) complex and signals via activation of the JAK-STAT pathway, ultimately causing transcription of interferon-stimulated genes (ISGs) [41,42]. ISGs include pro-apoptotic genes, such as TRAIL and Fas/CD95. IFNα also causes downregulation of cyclins and other cell cycle genes, resulting in G1 arrest. IFN-α is produced endogenously in response to TLR3 activation [41,42]. In addition to exerting effects directly on tumor cells, IFN-α links the innate and adaptive immune systems by enhancing T cell proliferation and survival, and NK cell cytotoxicity [27].

Below, we discuss IFN-α’s role in treatment of the main myeloid malignancies: the myeloproliferative neoplasms (chronic myeloid leukemia (CML), polycythemia vera (PV), essential thrombocythemia (ET), and primary myelofibrosis (PMF), as well as in acute myeloid leukemia (AML) and myelodysplastic syndromes (MDS). A list of recent clinical trials utilizing various formulations of IFN-α for MPNs, MDS, and AML can be found in Hemmati et al. [43].

### 3.1. MPNs

CML is a myeloproliferative neoplasm, characterized by a translocation event, resulting in the formation of the Philadelphia chromosome and expression of the BCR-ABL transgene. In the early 1980′s, IFN-α was found to exert anti-proliferative effects on myeloid progenitor cells [44], leading to the hypothesis that it could be used clinically for treatment of MPNs. IFN-α was one of the earliest treatments for chronic myeloid leukemia that was shown to prolong 5-year overall survival (OS) compared to conventional chemotherapy (50–59% with IFN-α compared to 29–44% with busulfan or hydroxyurea) [45,46,47]. One mechanism of interferon-induced CML cell death is thought to involve an increase in Fas receptor on CML progenitor cells [48]. Other proposed mechanisms include interferon-induced expansion of NK cells and γδ-T cells, as well as induction of cytotoxic T cell responses [49,50]. Thus, IFN-α was the standard frontline treatment for CML until the emergence of molecular targeting in the form of tyrosine kinase inhibitors (TKIs).

Despite the efficacy of TKIs in CML disease control, they are not curative in most cases, and therefore most patients require TKIs indefinitely to keep their disease in check. Additionally, some patients develop resistance to TKI therapy. In order to increase efficacy and sustainability of TKI treatment, IFN-α has been combined with imatinib in multiple clinical trials, such as the German CML-Study IV [51] and French ST157I Prospective Randomized Trial (SPIRIT) [52], reviewed by Talpaz et al. [53]. The SPIRIT trial was a prospective randomized trial for frontline treatment of chronic-phase CML that included over 600 patients in four arms: imatinib 400 mg, imatinib 600 mg, imatinib 400 mg + cytarabine, and imatinib 400 mg + pegylated interferon alpha2a (PegIFN-α2a) (90 αÌg weekly). Endpoints included molecular and cytogenetic responses, time to treatment failure, OS, event-free survival, and adverse events. Analysis at 12 months revealed a superior rate of molecular response in patients receiving imatinib plus PegIFN-α2a (30%) versus 400 mg imatinib alone (14%) [52]. Patients receiving PegIFN-α2a, however, had a high rate of discontinuation due to toxicity (45% within the first year), so the dose was reduced. The long-term results of the SPIRIT trial, which was started in 2003, were published in early 2021 [54]. The rate of major molecular response (BCR-ABL transcript level ≤ 0.1%) was higher in the imatinib + PegIFN-α2a group than imatinib 400 mg alone group (59% vs. 41%, respectively), but this difference did not translate into a difference in progression free survival (PFS) or OS. While the addition of IFN-α to a TKI in this study did not prolong PFS or OS, IFN-α may still have a role in CML treatment, e.g., in the maintenance of remission after TKI discontinuation [55]. There is a phase III trial in progress, comparing PegIFN-α2a to observation after stopping TKI, in CML patients who have had a deep molecular response for at least 2 years (NCT02381379). In fact, there are over 50 studies either active or completed that are studying various formulations of IFN-α treatment in CML [56].

PV and ET are myeloproliferative neoplasms that can cause symptoms and pose a risk of progression to acute myeloid leukemia. ET and PV patients are at risk for major bleeding, as well as thrombosis. Low-risk patients are managed with low-dose aspirin, with the addition of phlebotomy in the case of PV. Hydroxyurea is typically the agent of choice for cytoreduction in PV and ET patients, but it does not cause a cytogenetic remission. In ET and PV patients younger than age 65 with disease that is refractory to hydroxyurea, or in cases of intolerance or pregnancy, interferon can be used [57]. For example, one study of weekly IFN-α in PV patients reported a hematologic remission rate of over 80% [58]. A phase II study, that included both PV and ET patients, reported that 69% of ET patients and 100% of PV patients had a response (either PR or CR) to PEG-IFN-α-2b, though this was a small study that included 13 ET patients and only 4 PV patients [59]. A larger trial that included 39 ET patients and 40 PV patients, 81% of whom had received prior therapy, reported high overall hematologic responses (81% in ET and 80% in PV) to pegylated IFN-α [60]. Hematologic response was defined as normalization of platelet counts and absence of thromboembolic events in ET, as well as normalization in hematocrit in PV patients (50).

PMF is a myeloproliferative neoplasm associated with recurrent mutations in *JAK2* (most frequent), *CALR*, or *MPL*, which cause abnormal proliferation of myeloid cells and bone marrow fibrosis. This abnormal myeloid growth results in splenomegaly, constitutional symptoms, and abnormal cell counts. In some cases, PMF can progress to AML. A similar entity, secondary myelofibrosis, occurs in patients initially diagnosed with ET or PV. In some smaller studies, IFN-α has shown some promise for both PMF and secondary myelofibrosis treatment. The first study, suggesting benefits of IFN-α in the treatment of myelofibrosis, was published in 1987 [61]. This study included two symptomatic patients, one with post-PV myelofibrosis and one with PMF, who were treated with daily subcutaneous injections of recombinant IFN-α-2c. Both patients had a reduction in bone pain and improvement in splenomegaly, but they experienced severe cytopenias, requiring multiple dose reductions in IFN-α-2c. Ultimately, one of the patients had recurrence of splenomegaly at which point treatment was stopped. There have since been more promising reports of IFN-α treatment for primary and secondary myelofibrosis. For example, in one study, treatment with recombinant IFN-α, in “early” PMF patients, resulted in stable or improved disease in 14 out of 17 patients [62]. Another study looked retrospectively at patients with primary or secondary myelofibrosis who received at least 6 months of Peg-IFN-α-2a treatment, and found that 82% of patients had resolution of their constitutional symptoms, 38.5% achieved transfusion independence, and 82.8% achieved complete resolution of their thrombocytosis [63]. Despite some promising results with IFN-α treatment and the development of targeted agents (e.g., the JAK inhibitor ruxolitinib), allogeneic stem cell transplant remains the only curative treatment for PMF.

### 3.2. MDS and AML

MDS is a bone marrow disorder that is characterized by dysfunctional hematopoiesis, leading to cytopenias and variably increased blasts. While MDS cells have multiple genetic mutations and cytogenetic abnormalities, it is likely that immune dysfunction plays a role in the development of the disease as well. Evidence of this dysfunction includes a study looking at bone marrow-derived mesenchymal stem cells from 7 MDS patients (compared with healthy controls) that revealed an upregulation of IFN-α/β signaling and *ISG15* in the MDS-derived samples [64]. Another study found that several of the genes, upregulated in MDS patients’ CD34+ cells, were interferon-stimulated genes, such as *STAT1*, *IRF9*, interferon-induced protein with tetratricopeptide repeats 1 and 3 (*IFIT1* and *IFIT3*), interferon-induced transmembrane protein 1 (*IFITM1*), and interferon-induced protein 44-like (*IFI44L*) [65]. Additionally, IFN-γ is overexpressed in the bone marrow of MDS patients [66].

In AML, there are many mechanisms of immune evasion and suppression by the leukemia cells. These may include secretion of cytokines and other factors that alter the bone marrow niche, upregulation of inhibitory T-cell ligands, and expansion of regulatory T cells and myeloid-derived suppressor cells [67]. A recent study on IFN-α treatment for minimal residual disease (MRD) positive t(8;21) AML patients after allo-HSCT reported a 2-year OS of 92.3%, compared to 51.4% in historical cohorts [68]. Another recent study showed a benefit of IFN-α maintenance treatment in favorable-risk AML in reducing risk of relapse [69]. A pilot phase II study of GM-CSF and IFN-α-2b, in patients with relapsed disease (including AML, blast phase CML, and MDS) after allo-HSCT, was conducted (NCT00548847), and results are pending.

### 3.3. STING Agonists

Another strategy utilizing interferon’s anti-cancer properties is to stimulate endogenous IFN production, e.g., by activating the stimulator of interferon genes (STING) pathway. Activated STING causes interferon regulatory factor 3 (IRF3) to initiate transcription of type I IFNs as well as other cytokines [70]. In one preclinical model, the STING activator DMXAA showed STING-dependent anti-leukemic activity in a mouse model of AML [71]. There are ongoing preclinical studies of these agents in multiple tumor types (reviewed in [72,73]). There is currently a phase I/Ib clinical trial (NCT04144140) using the STING agonist E7766 in patients with advanced tumors or lymphomas. STING agonists are also being studied for use in conjunction with checkpoint inhibitors such as TAK-500 or TAK-676 with pembrolizumab (NCT05070247 and NCT04420884).

## 4. Antibody Therapy in AML

### 4.1. Unconjugated Antibodies

Unconjugated antibodies work by facilitating NK cell function via antibody-dependent cell-mediated cytotoxicity (ADCC). Currently, there are no unconjugated antibodies recommended in the NCCN guidelines for treatment of AML. There are, however, multiple such agents showing promising preclinical data, and some are currently in clinical trials. Some of these antibodies target the leukemia cells themselves, while others aim to block the leukemia cells from interacting with their microenvironment.

CD47 is a transmembrane protein that is overexpressed in multiple solid and hematologic malignancies, including AML. CD47 functions by binding to its cognate receptor called Signal Regulatory Protein Alpha (SIRPα) on macrophages and dendritic cells, leading to disruption of the phagocytic synapse site, and ultimately preventing phagocytosis of the cancer cell. Magrolimab (Hu5F9-G4) is an anti-CD47 monoclonal antibody that targets CD47 on AML blasts, restoring a functional macrophage immune checkpoint. This antibody has shown some efficacy in AML and MDS patients in early phase clinical trials. For example, a phase Ib trial of the hypomethylating agent azacitidine, in combination with magrolimab, in previously-untreated AML patients unfit for intensive chemotherapy reported an objective response rate (ORR) of 65%. Importantly, there was a 71% ORR in *TP53*-mutant AML patients, a traditionally difficult-to-treat population [74]. The ENHANCE clinical trial (NCT04313881) is an ongoing phase III trial comparing safety and efficacy of azacitidine plus magrolimab versus azacitidine plus placebo in previously-untreated patients with high-risk MDS. As of the writing of this review, there are at least five clinical trials involving magrolimab treatment in myeloid malignancies (clinicaltrials.gov), all in combination with azacitidine and/or the BCL-2 inhibitor venetoclax.

CD99 is a transmembrane protein that is frequently overexpressed on AML and MDS cells. Targeted monoclonal antibodies, against CD99, appear active against AML cells and xenografts in preclinical models [75,76]. Evidence suggests that the mechanism of action of anti-CD99 antibodies is via activation of SRC-family kinase (SFK), resulting in oncogenic stress, cell cycle arrest, and apoptosis of leukemia cells [75]. At the writing of this review, there are no clinical trials utilizing anti-CD99 antibodies in treatment of AML or MDS listed on public databases.

CD38 is a glycoprotein that is expressed on AML cells as well as plasma cells; anti-CD38 antibodies such as daratumumab and isatuximab are already approved in the treatment of multiple myeloma. These antibodies bind to CD38 and induce complement-mediated cytotoxicity, ADCC, antibody-dependent cell phagocytosis, and apoptosis. There are preclinical data using AML cell lines or patient-derived AML xenograft mouse models suggesting efficacy of anti-CD38 antibodies [77,78,79]. Clinical trials using daratumumab (NCT03067571, NCT03537599) and isatuximab (NCT03860844) in AML treatment are ongoing.

*FLT3* is a gene that encodes a receptor tyrosine kinase, and is the most frequently mutated gene in AML [80]. FLYSYN is a chimeric Fc-optimized IgG antibody targeted to FLT3 (CD135). A phase I trial (NCT02789254) using FLYSYN in AML patients with minimal residual disease reported good safety and tolerability, as well as a molecular response in 11/31 (35%) of patients, with an ORR of 46% in the highest dose arm [81]. An earlier phase I trial with a different FLT3 antibody, LY3012218 (IMC-EB10), showed no clinical activity in relapsed AML [82].

All of the antibodies discussed above target the AML cells directly. Another strategy for AML treatment involves using monoclonal antibodies to disrupt the interaction between leukemia cells and their microenvironment. Ulocuplumab (BMS-936564) is a human IgG4 monoclonal antibody against the G-protein coupled chemokine receptor CXCR4 that prevents it from binding to its ligand, the chemokine CXCL12 (also known as stromal cell-derived factor 1) [83]. When CXCR4 is blocked from binding to CXCL12, leukocytes mobilize from the bone marrow niche into the peripheral blood where they can be exposed to higher levels of chemotherapeutics [84]. Ulocuplumab also causes apoptosis of leukemia blasts ex vivo [84]. A phase I clinical trial, studying the safety and efficacy of ulocuplumab in 73 patients with relapsed/refractory AML in combination with mitoxantrone, etoposide, and cytarabine (MEC) reported an improvement in response rate with this novel combination (CR + CRi of 51% compared with the historical response rate of 24–28% with MEC alone [84].

The integrin-binding glycoprotein CD98 also plays an essential role in the proliferation of leukemia cells by engaging them with their microenvironment [85]. This knowledge led to the development of the anti-CD98 monoclonal antibody IGN523 and the phase I study of single-agent IGN523 in 19 adult patients with relapsed/refractory AML [86]. Transient anti-leukemic activity was seen in three patients, but no partial or complete responses were observed. Despite this lack of efficacy as a single agent, IMG523 may prove useful in combination with other leukemia-directed therapies. A summary of recent clinical trials, using unconjugated antibodies in AML treatment, can be found in Table 1.

### 4.2. Multivalent Antibodies

The purpose of using multivalent antibodies is to increase the immune response to tumor cells by physically approximating the two cell types. Bispecific T-cell engagers (BiTEs) are one type of multivalent antibody [87]. They are engineered to link two antibodies: one that targets the antigen of interest on the cancer cell, and one that is directed to the CD3 receptor on T cells. Once the BiTE is bound to CD3, the T cell receptor is stimulated and activates a cytotoxic response against the blast cell [88]. This specific interaction decreases the risk of off-target toxicity, as these T cells are only activated near blast cells. BiTE molecules are very small and can be rapidly excreted by the kidney; thus, the new generation of BiTEs are being designed to have a longer half-life [89]. There are various antigens of interest on blast cells that could theoretically serve as targets for BiTEs, however, it is important that the antigens meet certain criteria in order to serve as effective targets. For example, the antigens should have high expression on blast cells and low, or absent, expression on normal hematopoietic cells.

There are multiple BiTE therapies that have been developed and studied in AML. The first BiTE therapy developed in myeloid malignancies was against CD33 (AMG 330). The BiTE molecule binds to the IPYYDKN amino acid sequence within the CD33 V type domain on blast cells and engages it with CD3 on T cells, resulting in T cell activation, expansion, and cytotoxic killing. In a study by Ravandi et al., 55 patients with relapsed/refractory AML received AMG 330; the ORR was 19%, and CR was 7%. The main adverse event, experienced in 60% of patients, was cytokine release syndrome (CRS) [90]. Subklewe et al. used a newer generation CD33 BiTE (AMG 673) which has an extended half-life. In that study, 30 patients were enrolled, 44% of patients had a reduction in blast count in the bone marrow, and 50% of patients developed CRS [91].

Due to the increased expression of CD123 on AML cells, multiple BiTE therapies have been developed using that target. Ravandi et al. reported a study that evaluated the CD123-targeted BiTE ibecotamab in 103 relapsed/refractory AML patients [92]. The ORR in that study was 14%, and four patients had a CR. Interestingly, 71% of patients had stable disease. Of note, CRS was observed in 59% of the patients. Talacotuzumab (another CD123 BiTE) has been evaluated in a phase II/III study in combination with decitabine (compared to decitabine alone) [93]. This study enrolled 316 older AML patients that are ineligible for intensive chemotherapy. The combination therapy yielded OS and CR rates that are similar to single agent decitabine (median OS: 5.36 months with combination vs. 7.26 months for decitabine alone with HR: 1.04; 95% CI: 0.79–1.37; *p* = 0.78) (CR: 15% vs. 11%; odds ratio: 1.4; 95% CI: 0.6–3.6; *p* = 0.44). Flotetuzumab is a newer generation CD123 BiTE called a dual affinity retargeting antibody (DART) that consists of a diabody backbone with the addition of a c-terminal disulfide bridge that is aimed to increase stabilization of the molecule. In a study by Uy et al., flotetuzumab was evaluated in 30 patients with relapsed/refractory AML [94]. The ORR in that study was 27%, with a median OS of 10.2 months in the responders. This study, however, reported a 100% rate of CRS.

Given limited responses observed in targeting CD33 and CD123, other specific targets on AML blasts are being evaluated. For example, AMG 427 and BiTE 7370, which are novel anti-FLT3 x CD3 BiTEs, are being evaluated in relapsed/refractory AML [95,96]. In preclinical studies, treatment with AMG 427 causes an upregulation of PD-1 expression on T cells, decreasing the potency of AMG 427 against AML cells that express PD-L1; the addition of PD-1 blockade, however, restored AMG 427 potency in clearing blast cells [96]. In order to improve the limited response seen with BiTEs, multiple preclinical studies are evaluating the combination of FDA-approved therapies with BiTEs. One study evaluated the effect of adding venetoclax to CD123 BiTE therapy and showed that there was a dose-dependent blast reduction in mouse models in the combination arm compared to BiTE therapy alone [97].

Table 2 summarizes recent and ongoing clinical trials utilizing multivalent antibodies in the treatment of myeloid malignancies. Although a promising technology, so far, studies with multivalent antibodies have not shown major efficacy. Thus, the future utility of these agents may be dependent on combinations with other antileukemic agents.

### 4.3. Antibody-Drug Conjugates (ADCs)

ADCs are monoclonal antibodies that are conjugated to various cytotoxic agents via a linker molecule [98]. The target antigen for the ADCs is usually a relatively specific blast cell surface antigen with limited expression on healthy tissues [99]. The linker molecule is designed to stabilize the ADC in circulation and prevent the premature release of the attached cytotoxic agents, thereby maximizing the drug exposure of the target cells. Once the antibody is bound to the target receptor, the receptor-antibody complex is endocytosed, and ultimately, the cytotoxic agent is released inside the target cell. The cytotoxic agents, either chemotherapy or bacteria-derived toxins, usually target the DNA or microtubules, resulting in blast cell death [87].

Currently, the only FDA-approved ADC in AML is gemtuzumab ozogamicin (GO). This ADC is a humanized anti-CD33 monoclonal antibody that is conjugated to calicheamicin, which is a bacterial toxin that binds the DNA and causes strand scission [100]. GO was initially approved in 2000, based on its clinical efficacy in AML in a phase II study [101]. However, it was subsequently pulled from the market due to an increasing incidence of sinusoidal obstructive syndrome (SOS) and mortality [101,102]. Notably, GO was given without dose capping, and some patients who received GO subsequently underwent a stem cell transplant, which also increases the risk of SOS. After multiple studies, that evaluated lower doses of GO in combination therapy, showed efficacy with a much lower risk of SOS in patients with favorable cytogenetics, it was re-approved by the FDA [103]. It should be noted, however, that the agent has no significant activity in cases with poor cytogenetics [104].

There are multiple other CD33-directed ADCs. Actinium-225 (^225^Ac)-lintuzumab is a radioimmunoconjugate composed of ^225^Ac conjugated to a CD33 antibody. A single arm phase II study of ^225^Ac-lintuzumab in older patients with untreated AML reported preliminary results with a 56% ORR [105]. Further studies using ^225^Ac-lintuzumab, in combination with other treatments such as salvage chemotherapy or venetoclax, are planned [105]. Vadastuximab talirine (SGN-CD33A) is an ADC which incorporates a cytotoxic synthetic DNA crosslinking pyrrolobenzodiazepene dimer to CD33 [106]. One study evaluated this ADC in combination with azacitidine in the frontline setting for patients above age 60 and reported an overall response rate (CR/CRi) of 70% with high MRD negativity [107]. However, a subsequent randomized placebo-controlled study was discontinued due to increased mortality that is likely due to bone marrow suppression, as CD33 is expressed on normal hematopoietic stem cells and increased toxicity from the cytotoxic agent pyrrolobenzodiazepene. This toxicity highlights the critical need to identify other AML-specific targets (NCT02785900).

IMGN632 is an ADC that binds to a different target: CD123. CD123 is an IL-3 receptor alpha chain that is highly expressed on AML blasts, but also expressed on endothelial cells and hematopoietic stem cells [108]. IMGN632 links a CD123 antibody to an indolinobenzodiazepine pseudo-dimer. A phase I/Ib study, evaluating this ADC in 66 patients with AML, showed a response rate of 20% (4% CR, 12% CRi, 3% morphologic leukemia free state), which is the basis of a phase II study with HMA and venetoclax treatment in patients who are not candidates for intensive chemotherapy [109]. AGS 62P1 (ASP 1235) is an ADC that links a FLT3 antibody to AGL-0182–30, a microtubule-disrupting agent [109,110]. Early phase studies are showing potent cytotoxicity towards FLT3-mutated AML cells [111]. Finally, iodine-131 (^131^iodine) apamistamab (Iomab-B), an ADC that links a CD45 antibody to radiolabeled ^131^iodine, is being evaluated in patients who are unable to tolerate standard high-dose myeloablative HSCT pre-conditioning, is showing promising results and a decreased risk of mucositis, neutropenic fevers, and sepsis [112].

## 5. Checkpoint Inhibitors

Immune check point inhibitors (ICI), such as PD-1 and CTLA-4 antibodies, have transformed the treatment of many cancers, including some hematological malignancies [113]. However, thus far their efficacy in myeloid malignancies has been limited. Hypomethylating agents (HMAs) have efficacy in AML and MDS and are known to modify immune activation in several ways; however, they also increase immune checkpoints, leading to the idea of combining HMAs with ICIs [114]. One study that evaluated the use of the anti-PD-1 antibody nivolumab in combination with azacitidine in relapsed/refractory AML reported an ORR of 18% and median OS of 9.3 months; interestingly, the subset of patients who had not been previously exposed to HMAs had an overall response rate of 52% [115]. Another study evaluated the use of the anti-CTLA-4 antibody ipilimumab in 28 patients with relapsed hematological malignancies after stem cell transplant, 12 of which had AML. This study showed no response with the lower dose of ipilimumab (3 mg/kg), but there was an ORR of 55% in patients receiving the higher dose (10 mg/kg) [116]. Finally, another study evaluated ipilimumab, in 29 patients with MDS who failed a prior hypomethylating agent, and showed a median OS of 294 days (with censoring of transplanted patients) [117]. As with other cancer types, biomarkers will likely play a role in selecting the subset of patients who will respond to ICI. For example, in the nivolumab plus azacitidine study, those with a pre-existing T cell infiltration and higher CD3+, CD8+ lymphocyte percentage in the pre-treatment bone marrow had the best chance of response [115]. Clinical studies on checkpoint inhibitors in AML and other myeloid malignancies are summarized in Table 3.

## 6. CAR-T and CAR-NK Cells in AML

Chimeric antigen receptor (CAR) T-cell therapy is an exciting novel therapeutic approach that is showing promise in lymphomas and multiple myeloma [118]. Furthermore, CAR-T therapies have received US Food and Drug Administration (FDA) approval in relapsed/refractory acute lymphocytic leukemia with CAR-T directed against CD19 [119]. However, CAR-T trials are still in the early stages of development in AML/MDS. The two most studied targets for CAR-T therapies in myeloid malignancies are CD33 and CD123. NCT03795779 is a trial currently recruiting patients with relapsed and refractory myeloid malignancies for treatment with a CD33-CLL1 (C-type lectin molecule-1) compound CAR-T and results are awaited.

As discussed previously, CD123 is highly expressed on AML blasts, but it is also expressed on endothelial cells and hematopoietic stem cells [108]. As a result, trials that evaluated CD123 CARTs have encountered complications of capillary leak syndrome (CLS) in addition to CRS, especially in patients with heavy disease burden [120]. To prevent these life-threatening adverse events, newer trials are using biodegradable CD123 CAR-Ts that are manufactured by electroporation of mRNA encoding the CAR, rather than T cells transduced with lentivirus, resulting in transient (rather than permanent) expression of the chimeric antigen receptor [121]. While this approach should decrease the persistence of CAR-Ts and, therefore, the risk of CLS, it will likely require repeated dosing [122]. Another issue with targeting CD123 is that, because it is present on normal HSCs, healthy HSCs may be destroyed during treatment. Therefore, patients who receive CD123 CAR-T may need a rescue allogenic stem cell transplant to repopulate their hematopoietic stem cell compartment. For example, a second generation CD123 CAR-T (CD123CAR-41BB-CD3ζ) trial (NCT03766126) plans to use allo-HSCT as a rescue strategy if patients experience prolonged marrow aplasia. Given these challenges in CAR-T therapies in myeloid malignancies, there are extensive efforts to find new targets; NKG2D, ADGRE2, CCR1, CD70, and LILRB2 are promising targets [123]. In addition, there is interest in developing dual CAR-CAR-T (CAR-T with 2 CAR antigens) or combination therapies of two different CAR-Ts [124,125].

NK cells, as discussed above, are part of the innate immune system, and they are able to recognize the absence of certain proteins that may be downregulated on malignant cells, such as HLA proteins. They also have the ability to kill tumor cells directly. Thus, NK cells can be engineered to target cancer cells, such as CAR-T cells. In fact, CAR-NK cell therapies are emerging as a promising new treatment [126]. Unlike CAR-T cells, CAR-NK cells do not carry the risk of graft-versus-host disease (GvHD) and, therefore, could be engineered as an off-the-shelf product that would be readily available for immediate clinical use [127]. Recently, the first in human phase I study with CAR-NK was reported and involved three patients with relapsed/refractory AML treated with anti-CD33 CAR NK-92 [128]. These NK-92 CARs are third-generation CARs that incorporate both CD28 and 4–1BB co-stimulatory molecules. This study established the safety of escalating doses of CAR-transduced NK-92 cell infusions; however, no durable responses were achieved. NCT02944162 is the phase II study that will evaluate the efficacy of NK-92 CAR with the highest dose. Currently, there is a phase I study evaluating NKX101 CART which are allogeneic CAR-NK cells targeting NKG2D ligands in patients with relapsed/refractory AML and high risk MDS (NCT04623944). The NK cells are derived either from haplo-matched related donors or unrelated off-the-shelf donors.

## 7. Discussion

The immune system plays an essential role, in preventing tumor formation, by recognizing and killing cells that have become altered from normal “self.” Although the immune system has redundant mechanisms of surveilling for tumors, neoplastic cells are able to suppress or subvert these mechanisms. Manipulation of the immune system, however, is a strategy that is growing increasingly effective against malignancies. From interferon, which has been used to treat myeloid malignancies for decades, to the promise of CAR-T and CAR-NK cells, immuno-oncology appears to be a longstanding fixture in the treatment of myeloid malignancies. Each of these strategies has its own set of limitations and side effects. Most immune-mediated strategies require the targeting of an AML antigen; however, it is difficult to find tumor antigens that are unique to the AML cells, and the downregulation of these antigens by the tumor cells is a common mechanism of resistance. Additionally, all immunotherapy-based strategies present a risk of autoimmune or inflammatory complications in patients, as well as a high risk of graft versus host disease in patients that have relapsed post-allo-HSCT. Despite the numerous immunotherapy agents mentioned above, few have been successful in clinical trials, which may be due, in part, to biological mechanisms of resistance that are yet to be delineated. Additionally, it will be important to continue identifying biomarkers for response to immunotherapy.

## Figures and Tables

**Table 1:** Ongoing and recently completed clinical trials of unconjugated antibodies in AML or high-risk MDS. MEC, mitoxantrone, etoposide, and cytarabine. DLI, donor lymphocyte infusion. AZA, azacitidine. Ven, venetoclax. MRD, minimal residual disease.

Drug	Target	Drug Combination	Phase	NCT Number	Patient Population
**Magrolimab**	CD47	AZA	III	NCT04313881	Previously untreated high-risk MDS
		AZA + Ven	III	NCT05079230	Newly diagnosed AML ineligible for intensive chemotherapy
		AZA	III	NCT04778397	Newly diagnosed TP53 mutant AML
**AK117**		AZA	I/II	NCT04980885	AML without favorable risk cytogenetics
**Daratumumab**	CD38	N/A	II	NCT03067571	R/R AML or high-risk MDS
		DLI	I/II	NCT03537599	Relapsed AML after stem cell transplant
**Isatuximab**		Chemotherapy	II	NCT03860844	Pediatric patients with R/R ALL or AML in first or second relapse
**FLYSYN**	FLT3 (CD135)	N/A	I	NCT02789254	AML patients with MRD
**PF-04518600** **(OX40)**	CD134	AZA, ven, glasdegib, avelumab, GO	I/II	NCT03390296	R/R AML
**Hu5F9-G4**	CD47	Atezolizumab	Ib	NCT03922477	R/R AML
**Talacotuzumab**	CD123	Decitabine	II/III	NCT02472145	AML ineligible for intensive chemotherapy
**Cusatuzumab**	CD70	Ven	Ib	NCT04150887	Previously-untreated AML ineligible for intensive chemotherapy
**IO-202**	LILRB4	N/A	I	NCT04372433	R/R AMML and CMML

**Table 2:** Recent clinical trials of multivalent antibodies for AML. All trials are for adults only. TriKE, tri-specific killer cell engager. R/R, relapsed/refractory. MRD, minimal residual disease. DART, dual-affinity retargeting antibody.

Drug	Targets	Phase	NCT Number	Patient Population	Drug Type
**MCLA-117**	CLEC12A × CD3	I	NCT03038230	-R/R AML -newly diagnosed AML in elderly patients with high-risk cytogenetics -very high risk MDS with R/R disease	Bispecific antibody
**AMG 330**	CD33 × CD3	I	NCT02520427	-R/R AML -MRD + AML -MDS	BiTE
**AMG 673**		I	NCT03224819	R/R AML	BiTE
**GEM333**		I	NCT03516760	CD33+ AML: R/R, or not eligible for standard induction therapy and refractory to or progressive after HMAs	BiTE
**JNJ-67371244**		I	NCT03915379	-R/R AML -high or very high risk MDS	BiTE
**Vibecotamab (XmAb14045)**	CD123 × CD3	I	NCT02730312	-Primary or secondary AML -B-ALL -BPDCN -Blast phase CML resistant or intolerant to TKIs	BiTE
**JNJ-63709178**		I	NCT02715011	R/R AML	DuoBody
**Flotetuzumab**		I/II	NCT02152956	Primary induction failure/early relapsed AML	DART
**Talacotuzumab** (+ decitabine vs. decitabine alone)		II/III	NCT02462145	AML not eligible for standard induction therapy	BiTE
**GTB-3550**	CD16 × IL-15 × CD33	I/II	NCT03214666	CD33+: -R/R AML -High-risk MDS -advanced systemic mastocytosis	TriKE
**LAVA-051**	CD1d × TCR	I/II	NCT04887259	R/R CD1d+ CLL, MM, and AML	BiTE
**AMG 427**	FLT3 × CD3	I	NCT03541369	R/R AML	BiTE

**Table 3:** Clinical trials using checkpoint inhibitors for the treatment of myeloid malignancies. T-AML, therapy-related AML. R/R. relapsed/refractory. DLI, donor lymphocyte infusion. MPAL, mixed phenotype acute leukemia. CMML, chronic myelomonocytic leukemia. AZA, azacitidine.

Drug	Target	Phase	NCT Number	Patient Population	Intervention
**Ipilimumab**	CTLA-4	I	NCT02890329	R/R MDS/AML	Ipilimumab + decitabine
		I	NCT03912064	Relapsed AML, MDS, or MPN after allo-HSCT	Ipilimumab + CD25/Treg-depleted DLI
**Durvalumab**	PD-L1	I	NCT02117219	MDS	Durvalumab + AZA, durvalumab + tremelimumab
		II	NCT02775903	High risk MDS, elderly AML patients	Durvalumab + AZA
**Pembrolizumab**	PD-1	I	NCT04284787	AML, t-AML, AML with MRC	Pembrolizumab + induction chemo or venetoclax + AZA
		I	NCT02981914	Hematologic malignancy with relapse after allo-HSCT	Pembrolizumab
		I	NCT03286114	Relapsed MDS/AML	Pembrolizumab
		I	NCT03969446	Newly diagnosed AML, MDS	Pembrolizumab + Decitabine
		II	NCT02768792	R/R AML	Pembrolizumab following HiDAC salvage induction
		II	NCT02845297	R/R MDS/AML and newly diagnosed AML patients >65	AZA + pembrolizumab
		II	NCT02996474	R/R AML	Pembrolizumab + decitabine
		II	NCT02708641	AML >60 in remission and not transplant candidates	Pembrolizumab
		II	NCT02771197	AML patients with high risk of relapse	Pembrolizumab + Fludarabine/melphalan + auto-HSCT
**Nivolumab**	PD-1	II	NCT03600155	R/R AML after HCT	Ipilimumab, Nivolumab
		II	NCT02275533	Post remission AML	Nivolumab
		II	NCT02532231	AML with high risk of relapse	Nivolumab
		I/II	NCT02464657	AML/MDS	Nivolumab & 7+3 induction
		II	NCT02397720	MDS/RR-AML, MPAL, CMML	Nivolumab + AZA +/− ipilimumab
		I	NCT02846376	AML & MDS after SCT	Nivolumab + ipilimumab
		I	NCT01822509	Hematologic malignancy with relapse after allo-HSCT	Nivolumab or Ipilimumab
		II	NCT04913922	R/R-AML and patients ≥ 65 with newly diagnosed AML	AZA + nivolumab + relatlimab [Anti-LAG3]
		II/III	NCT03092674	Elderly patients with MDS or newly diagnosed AML	AZA +/− nivolumab or midostaurin, or decitabine + cytarabine
**MBG453**	TIM-3	I	NCT03066648	AML/MDS	MBG453 + decitabine, or + PDR001 (anti-PD-I antibody)

## Data Availability

Not applicable.
